# Effect of the Oral Administration of Common Evening Primrose Sprout (*Oenothera biennis* L.) Extract on Skin Function Improvement in UVB-irradiated Hairless Mice

**DOI:** 10.3390/ph14030222

**Published:** 2021-03-06

**Authors:** Hoon Kim, Soon Yeong Park, Dae Kyun Chung

**Affiliations:** 1 Skin-Biotechnology Center, Kyung Hee University, Gwanggyo-ro 147, Yeongtong-gu, Suwon 16229, Korea; 2 Dain Natural Co., Ltd. 130-33, Gasong-ro, Pungse-myeon, Dongnam-gu, Cheonan 31216, Korea; rnd@dainnatural.com; 3 Graduate School of Biotechnology, Kyung Hee University, Deogyeong-daero 1732, Giheung-gu, Yongin 17104, Korea

**Keywords:** *Oenothera biennis* sprout, skin aging, skin function improvement, nutricosmetic, functional food

## Abstract

Most of the studies on common evening primrose (*Oenothera biennis* L.) are focused on its oils (isolated from seed, root, and stem tissues). We aimed to investigate the effect of the oral administration of OBS-E on the improvement of skin function in skin-damaged hairless mice exposed to excessive ultraviolet B (UVB) radiation owing to the preliminary in vitro findings regarding the antioxidant, anti-wrinkle, and skin moisturizing activities of OBS-E. OBS-E administration for 14 weeks did not significantly affect the body weight or clinical signs. Significant reductions were observed in wrinkle parameters (area, number, length, and depth, and metalloproteinase levels) in OBS-E-administered mice compared with those in UVB-irradiated control mice. OBS-E significantly increased skin elasticity and hyaluronic acid content, but it significantly decreased transepidermal water loss. Histomorphometrical analysis revealed that OBS-E significantly reduced the epidermal thickness, area of the collagen-occupied region, and number of microfolds and inflammatory and mast cells. These results demonstrate that OBS-E can effectively enhance skin functions in terms of ameliorating wrinkle formation, promoting skin-moisturization, enhancing skin barrier function, and inhibiting inflammatory reactions. The obtained results provide good starting point for the continuation in the process of developing new inner beauty products based on OBS-E.

## 1. Introduction

In recent decades, there has been an increased interest in delaying aging in the scientific and industrial fields. As one of the signs of human aging, skin aging, which can be easily visualized, provides clear evidence of the passing time, and is regarded as a complex biological process influenced by intrinsic (endogenous) and extrinsic (exogenous) factors [1]. Since ancient times, numerous plants and their extracts have been recognized as major sources for preventing skin aging in humans [2]. In addition to the cosmetic application of these molecules, they have ‘nutraceutical,’ ‘nutricosmetic,’ and ‘cosmeceutical’ effects, which serve as a means of improving skin function and inhibiting skin aging via oral administration [3,4]. Many researchers have investigated the novel functional agents present in natural substances.

Evening primrose, belonging to the genus *Oenothera*, is a biennial herbaceous plant with yellow flowers [5]. Though it is native to Southern America, approximately 145 species have been identified in other continents, such as Asia, Europe, Africa, and Oceania [6]. The most familiar and widely studied species of the *Oenothera* family is *Oenothera biennis* L., also known as the common evening primrose. Various physiological and medicinal properties of *O. biennis* have been reported, including antioxidant [7,8,9], anti-inflammatory [10,11], anti-cancer [12], anti-diabetes [13], anti-bacterial [14], hypocholesterolemia [15], anti-thrombosis, and anti-caries [16]. Various functionalities of *O. biennis* have been reviewed by Munir, et al. [17]. However, most of the studies on the physiological properties of *O. biennis* have been conducted using oils isolated from its seed, root, and stem tissues. In this context, we focused on the sprout of *O. biennis* (OBS) to investigate its potential application as a nutricosmetic agent. In our preliminary study, an ethanol extract of *O. biennis* sprout (OBS-E), containing two flavone glycosides (luteolin 7-*O*-glucuronide and quercetin 3-*O*-glucuronide) and two polyphenolic acids (gallic acid and ellagic acid), was prepared and its physiological effects on the skin were evaluated in terms of antioxidant, anti-wrinkle, and skin moisturizing enhancement effects using human keratinocytes and fibroblasts [18]. Results from this preliminary study demonstrated that OBS-E may serve as a functional ingredient in anti-aging and moisturizing products. However, despite the promising findings, the preliminary study had limitations owing to the lack of in vivo data. Thus, the aim of the present study was to investigate the in vivo effect of the oral administration of OBS-E on skin function improvement, to obtain the fundamental data for developing novel inner beauty products based on OBS-E. For this, low and high doses of OBS-E were administered for 14 weeks to photoaged hairless mice exposed consistently to ultraviolet B (UVB) irradiation, which acted as skin-damaged models, and various skin function-related parameters, such as wrinkle formation, skin elasticity and hydration, inflammatory mediators, and histomorphometrical parameters were investigated in the damaged skin tissue.

## 2. Results

### 2.1. Observation of Body Weight Change and Clinical Signs

The changes in body weight throughout the study are shown in Supplementary Figure S1. No significant changes in body weight were observed among the groups, suggesting that it was unaffected by OBS-E administration during the study period. In addition, no abnormal clinical signs or death were observed in any of the animals (data not shown).

### 2.2. Effect of OBS-E Administration on Wrinkle Amelioration

**Figure 1:**
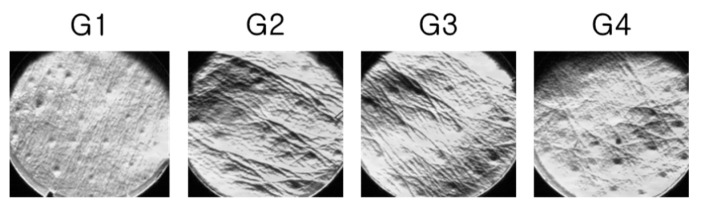
Photograph of the replica prepared from the dorsal skin of UVB-irradiated mice. (**G1**), non-irradiated control group administered saline; (**G2**), UVB-irradiated control group administered saline; (**G3**) and (**G4**), UVB-irradiated test agent groups administered OBS-E at doses of 50 and 200 mg/kg BW/day, respectively.

The replica images of the dorsal skin are provided in Figure 1. 

**Figure 2:**
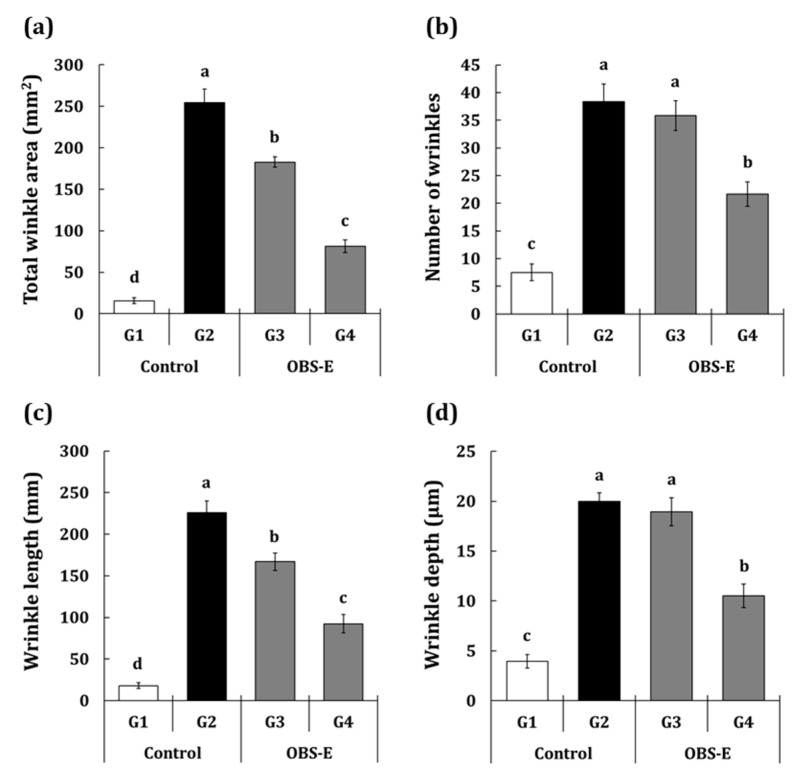
Effect of oral administration of OBS-E on wrinkle formation. (**a**) total wrinkle area, (**b**) wrinkle number, (**c**) wrinkle length, and (**d**) wrinkle depth. G1, non-irradiated control group administered saline; G2, UVB-irradiated control group administered saline; G3 and G4, UVB-irradiated test agent groups administered OBS-E at doses of 50 and 200 mg/kg BW/day, respectively. Different superscripts indicate significant difference among groups by Duncan’s multiple range test (*p* < 0.05).

Wrinkle formation was increased in the G2 group compared with that in the G1 group, whereas it was decreased in the test article-administered groups (G3 and G4) compared with that in the G2 group. Based on the replica images, the wrinkle factors, such as area, number, length, and depth were analyzed and presented in Figure 2a–d. Compared with the G1 group, all wrinkle factors, such as the wrinkle area (+1524.1%), number (+411.1%), length (+1153.4%), and depth (+407.0%) significantly increased in the G2 group. The G3 group showed a significant reduction in the total wrinkle area (−30.1%), length (−28.2%), and depth (−28.2%), whereas the wrinkle number and depth did not exhibit a significant difference, compared with those of the G2 group. In the G4 group, all factors analyzed, i.e., the wrinkle area (−72.6%), number (−54.1%), length (−64.2%), and depth (−59.1%) were significantly reduced compared with those in the G2 group.

**Figure 3:**
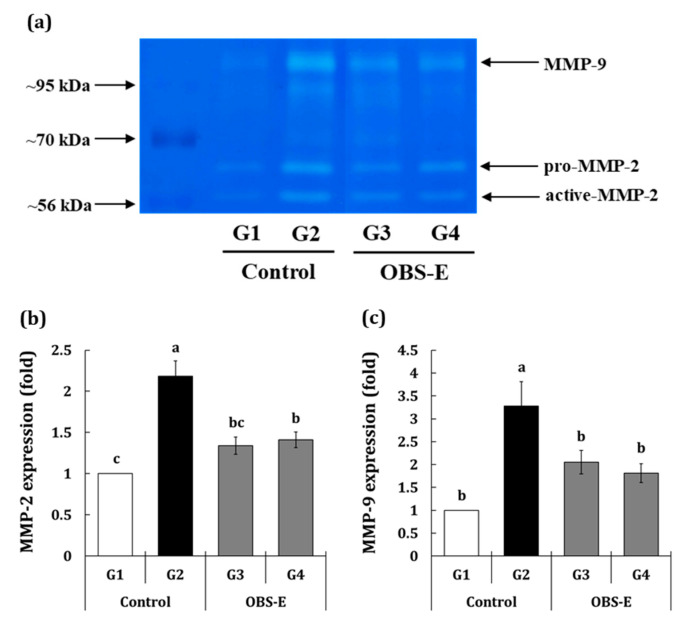
Effect of oral administration of OBS-E on the expression of matrix metalloproteinase (MMPs). (**a**) Gelatin zymographic images, (**b**) MMP-2 expression, (**c**) MMP-9 expression. G1, non-irradiated control group administered saline; G2, UVB-irradiated control group administered saline; G3 and G4, UVB-irradiated test agent groups administered OBS-E at doses of 50 and 200 mg/kg BW/day, respectively. Different superscripts indicate significant difference among groups by Duncan’s multiple range test (*p* < 0.05). Different superscripts indicate significant difference among groups by Duncan’s multiple range test (*p* < 0.05).

The expression of the wrinkle formation-related enzymes matrix metalloproteinase-2 (MMP-2) and matrix metalloproteinase-9 (MMP-9) is illustrated in Figure 3a–c. The expression of both factors was significantly higher in the G2 group (118.4 and 227.7%, respectively) than that in the G1 group. The G3 (−71.4 and −53.8%) and G4 (−65.4% and −64.3%) groups exhibited a significant decrease in MMP-2 and MMP-9 expression, respectively, compared with the G2 group. 

### 2.3. Effect of OBS-E Administration on Skin Hydration

**Figure 4:**
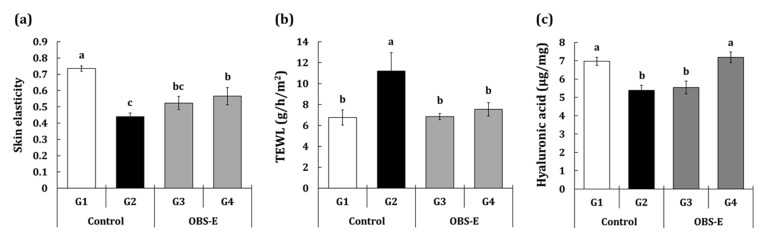
Effect of oral administration of OBS-E on (**a**) skin elasticity, (**b**) Transepidermal water loss, and (**c**) hyaluronic acid. G1, non-irradiated control group administered saline; G2, UVB-irradiated control group administered saline; G3 and G4, UVB-irradiated test agent groups administered OBS-E at doses of 50 and 200 mg/kg BW/day, respectively. Different superscripts indicate significant difference among groups by Duncan’s multiple range test (*p* < 0.05). Different superscripts indicate significant difference among groups by Duncan’s multiple range test (*p* < 0.05).

The levels of skin elasticity, transepidermal water loss (TEWL), and hyaluronic acid HA) are provided in Figure 4a–c. Upon corneal analysis, significant differences were observed in skin elasticity (−40.2%) and TEWL (+65.8%) in the G2 group compared to those in the G1 group. The HA content analyzed using ELISA was significantly lower by 22.7% in the G2 group compared with that in the G1 group. The G3 group exhibited a significant difference in only TEWL (−98.2%). Whereas, significant differences were observed in all parameters, including skin elasticity (+42.6%), TEWL (−82.4%), and HA (+113.8%) for the G4 group, compared with the G2 group.

### 2.4. Effect of OBS-E Administration on Microfold Number, Epidermal Thickness, and Collagen-Occupied Region Area

**Figure 5:**
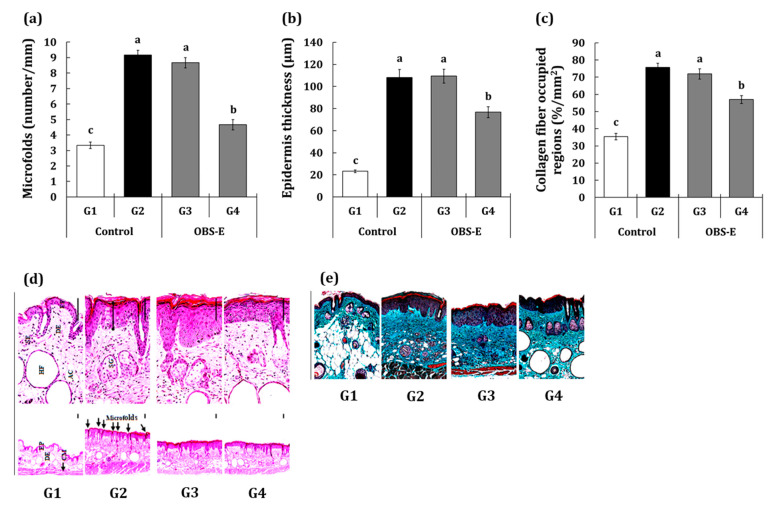
Effect of oral administration of OBS-E on (**a**) number of microfolds, (**b**) epidermis thickness, and (**c**) collagen fiber-occupied regions. (**d**) Skin biopsy samples stained with H&E for analyzing the microfold number and epidermal thickness, (**e**) Skin biopsy samples stained with Masson’s Trichrome stain for analyzing the collagen fiber-occupied region. AC, adipocyte; CM, cutaneous muscle; DE, dermis; EP, epidermis; EpTh, epidermal thickness measured; HF, hair follicle; SC, sebaceous gland; Scale bars = 80 μm. G1, non-irradiated control group administered saline; G2, UVB-irradiated control group administered saline; G3 and G4, UVB-irradiated test agent groups administered OBS-E at doses of 50 and 200 mg/kg BW/day, respectively. Different superscripts indicate significant difference among groups by Duncan’s multiple range test (*p* < 0.05).

Upon hematoxylin and eosin (H&E) staining of the skin biopsy, microfold numbers and epidermal thickness were analyzed and are shown in Figure 5a–c. Significant increases in microfold numbers (+175.0%) and epidermal thickness (+364.4%) were observed in the G2 group compared with those in the G1 group. Although the G3 group did not exhibit a significant decrease in both factors compared to the G2 group, microfold number (−77.1%) and epidermal thickness (−36.9%) in the G4 group were significantly lower than those in the G2 group. Collagen fiber-occupied regions were analyzed in skin biopsy samples stained using the Masson’s trichrome stain (Figure 5d,e). Similar to the results of H&E staining, the area of the collagen fiber-occupied region was significantly increased in the G2 group (+113.5%) compared with that in the G1 group, whereas only the G4 group (−46.5%), and not the G3 group, showed a significant reduction compared with the G2 group.

### 2.5. Effect of OBS-E Administration on the Number of Inflammatory and Mast Cells

**Figure 6:**
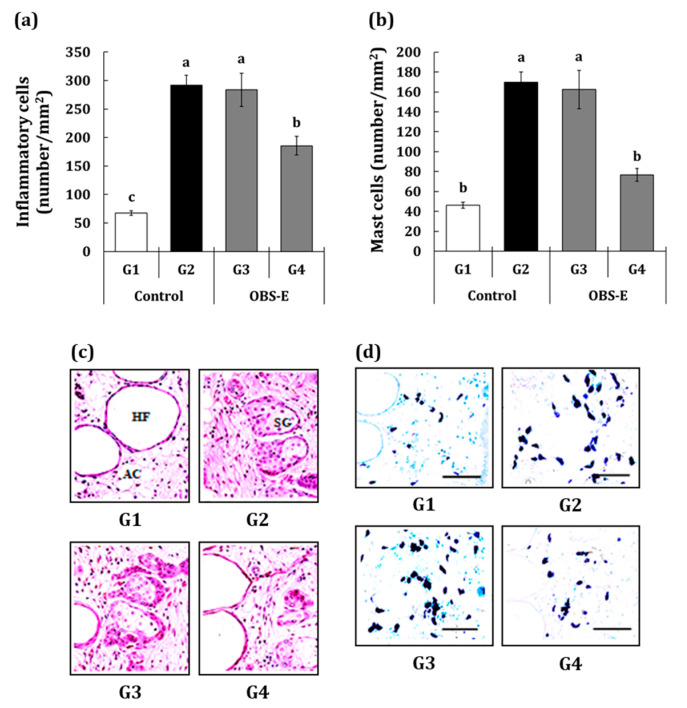
Effect of oral administration of OBS-E on the number of (**a**) inflammatory cells and (**b**) mast cells. (**c**) Skin biopsy samples stained with H&E for determining inflammatory cell counts, (**d**) skin biopsy samples stained with Toluidine blue for determining mast cell counts. HF, hair follicle; SC, sebaceous gland; Scale bars = 80 μm. G1, non-irradiated control group administered saline; G2, UVB-irradiated control group administered saline; G3 and G4, UVB-irradiated test agent groups administered OBS-E at doses of 50 and 200 mg/kg BW/day, respectively. Different superscripts indicate significant difference among groups by Duncan’s multiple range test (*p* < 0.05).

Inflammatory cells and mast cells were analyzed in skin biopsy samples stained with toluidine blue (Figure 6a–c). Significant increases in the number of both cells were observed in the G2 group (+334.7% and +266.2%, respectively) compared with those in the G1 group. Compared with the G2 group, the G3 group did not exhibit a significant difference in both cells, while significant decreases in both cells were observed in the G4 group (−47.3% and −75.4%, respectively).

## 3. Discussion

Skin is the largest organ in the human body, and the interest in maintaining healthy skin has been regarded as one of the important facets of social and private activities of humans [19]. To maintain healthy skin, the concept of ‘inner beauty’ is gaining increasing attention in recent years in addition to the use of cosmetics for outer beauty. Nutricosmetics is a concept that covers both beauty and nutrients, and it is developed to enhance external beauty or to care for skin health by oral consumption of foods, beverages, or supplements [4]. Since ancient times, many botanical extracts have been used as inner beauty products for healthy skin. We recently demonstrated via in vitro experiments the effects of OBS-E—mainly composed of gallic acid, luteolin 7-glucuronide, quercetin 3-glucuronide, and ellagic acid—on wrinkle amelioration and skin-moisturization. In this study, we aimed to investigate whether these effects can be observed in photoaged hairless mice. 

Administration of OBS-E at a concentration of 50 or 200 mg/kg BW once a day for 14 weeks was well-tolerated and no abnormal signs or significant changes in body weight changes were observed, thus indicating that the administration schedule and doses were safe. These results are consistent with those of a previous report which reported that *O. biennis* administration is not associated with any toxicity issues [20]. Significant differences were observed in all parameters measured for the UVB-irradiated control group compared with those for the non-irradiated control group, demonstrating that our UVB irradiation process induced damage in the mice. This suggests that our UVB-irradiated animal model is applicable for evaluating the efficacy of the test agents at healing damaged skin [19].

Compared with the non-irradiated controls, mice administered OBS-E exhibited improvement in wrinkle parameters, such as area, number, length, and depth, in a dose-dependent manner. In particular, a significant decrease in all parameters was observed for the group administered OBS-E at a dose of 200 mg/kg BW/day, thus suggesting its high ameliorative effect with respect to photoaging-induced wrinkle formation. In addition, the oral administration of OBS-E at 50 and 200 mg/kg BW significantly inhibited the expression of gelatinases (MMP-2 and MMP-9), which are responsible for the degradation of collagen fragments in the extracellular matrix of the skin tissue. As it is well known that consistent UVB exposure induces the expression of MMPs followed by the inhibition of procollagen synthesis, resulting in the degradation of the collagen matrix of the skin [21,22], our results indicate that orally administered OBS-E reduces wrinkle formation in the skin tissue damaged by photoaging and that the activity may be caused by downregulation of MMP-2 and MMP-9. Although studies have evaluated the wrinkle ameliorating MMP inhibitory effects of collagen peptide [23] and hydrolyzed chicken sternal cartilage extract (BioCell Collagen^®^; Biocell Technoloy, Irvine, CA, USA) [19], to our knowledge, this is the first report wherein the wrinkle ameliorating effect of *O. biennis* has been investigated in vivo.

Water is an essential component of the skin tissue, and skin hydration is one of the prerequisites for preventing skin aging and disease. The water holding capacity (barrier to water loss) of the stratum corneum plays a crucial role in maintaining the barrier function of the skin [24]. HA, which is mainly synthesized in dermal and epidermal tissue of the skin, is responsible for holding the water molecules and maintaining barrier function [25]. With respect to skin hydration, the oral administration of OBS-E resulted in significant changes in the elasticity, TEWL level, and HA content of the skin tissue, compared with those observed in UVB-irradiated controls. Particularly, OBS-E administration at a concentration of 200 mg/kg BW resulted in significant improvements in all parameters measured, demonstrating its effects in enhancing the efficacy of skin hydration, which was decreased upon UVB irradiation. However, for clearly elucidating the mechanism underlying OBS-E‒induced moisturizing efficacy, further results regarding the regulation of HA synthesis-related enzymes, such as hyaluronic acid synthases and hyaluronidases, are needed in the future studies.

As one of the characteristics of UVB-irradiation‒induced skin damage, the microfold number, epidermal thickness, and collagen-occupied region area are increased and can be observed by histological staining [26]. In particular, it is known that degradation of the extracellular matrix (ECM) by MMPs leads to the deposition of dense collagen followed by the expansion of regions occupied by non-cross-linked collagen fibers [19,27,28]. Histomorphometrical observations revealed that the UVB-irradiation‒induced increase in microfold number, epidermal thickness, and collagen-occupied region areas were decreased in the group administered OBS-E at a dose of 200 mg/kg BW.

UVB induces the accumulation of free radicals and reactive oxygen species, resulting in oxidative stress of cells, followed by damage and disease of skin tissue [29]. At this time, infiltration of pro-inflammatory cells, such as neutrophils and mast cells, is increased in the damaged skin tissue resulting in the generation of inflammatory responses for tissue repair [30]. Inflammatory cells also trigger the degradation of ECM via the secretion of various enzymes, resulting in various effects associated with photoaging [19,30]. Our results revealed a significant reduction in inflammatory cell and mast cell numbers in UVB-damaged lesions of the group administered OBS-E at high doses compared with those in the UVB-irradiated control group, thus confirming the anti-inflammatory effect of OBS-E during photoaging. Although some studies demonstrating the anti-inflammatory activity of *O. biennis* have been reported [17], this is the first study to definitely reveal the in vivo anti-inflammatory activity of *O. biennis*. Nonetheless, to elucidate the anti-inflammatory activity of *O. biennis*, further investigations on the levels of pro-inflammatory cytokines, such as interleukin (IL)-1, IL-6, IL-8, tumor necrosis factor alpha, and IL-10 in UVB-damaged skin lesions are needed.

## 4. Materials and Methods

### 4.1. Preparation of O. biennis Sprout Extract

The method used for the preparation of OBS-E has been presented in our recent published article [18]. Briefly, the ethanol extract of *O. biennis* sprouts was prepared in 50% ethanolic water by stirring at room temperature for 24 h, followed by filtration, evaporation, and drying. Our preliminary results revealed the presence of four major phytochemicals, gallic acid, ellagic acid, quercetin 3-glucuronide, and luteolin 7-glucuronide in OBS-E.

### 4.2. Animal Experiments

#### 4.2.1. Animals

The animal experiments were approved by the Chemon’s Animal Ethics Committee (IACUC No. 2020-02-005) and were performed in accordance with the Animal Research report of In Vivo Experiments (ARRIVE) guidelines [31]. Five-week‒old female specific pathogen-free hairless mice (Hos^®^: HR-1) were supplied by Joongah Bio (Gyeonggi, Republic of Korea). After acclimation for 7 days, the study was conducted in 24 animals. All animals were uniquely identified by marker pen and housed individually in polycarbonate cages (170 × 235 × 125 mm) in a room with controlled automatic ventilation (10 to 20 times/h) and lighting (12 h light/dark cycle). The temperature of the room and humidity were maintained at 23 ± 3 °C and 55 ± 15 %, respectively (values were recorded hourly throughout the study). Common rodent diet (Teklad-certified irradiated global 18% protein; DooYeol Biotech, Seoul, Republic of Korea) and sterilized water were provided ad libitum. 

#### 4.2.2. UVB Irradiation and Test Agent Administration

The test article (OBS-E) was dissolved in sterilized saline (Dai Han Pharm. Co., Ltd., Seoul, Republic of Korea). The mice were randomly divided into four groups (six mice per group). The control group (G1) was administered vehicle (sterilized saline) in the absence of UVB irradiation. The mice in the second group (G2) were administered vehicle and irradiated with UVB. The mice in the third and fourth groups (G3 and G4) were administered OBS-E with 50 mg/kg and 200 mg/kg, respectively, and irradiated with UVB. All animals were monitored daily throughout the test period for mortality and any changes in clinical signs and behavior; body weights and food consumption were recorded once a week throughout the study period. All animals were administered the test article or vehicle at a constant dose of 10 mL/kg by oral gavage. The mice in the G2, G3, and G4 groups were exposed to UVB (290 to 320 nm) three times per week throughout the study period. The workflow for UVB irradiation, which gradually increased from 60 mJ/cm^2^ to 180 mJ/cm^2^ throughout the experiment, has been described in a previous study [19].

### 4.3. Measurement of Skin Wrinkle, TEWL, and Elasticity

At the end of the experiment, all animals were euthanized by intraperitoneal injection of a mixture of zoletil (Virbac Carros, France) and rompun (Bayer Korea Ltd., Seoul, Republic of Korea). Skin elasticity and TEWL were analyzed using a Cutometer (Dual MPA 580; CK Electronic GmbH, Köln, Germany) and a Corneometer (GPSkin Barrier; GPSkin, Seoul, Republic of Korea), respectively, and the results were expressed as arbitrary units and g/h/m^2^, respectively. Skin replicas were prepared from UVB-irradiated dorsal skin tissues using the Visioline Replica Full Kit (CK Electronic GmbH), and wrinkle parameters; i.e., wrinkle area and number, depth, and length, of the replicas were analyzed using a wrinkle analyzing machinery (Visioline VL 650; CK Electronic GmbH). 

### 4.4. Histological Analysis

Paraffin blocks and tissue specimens from UVB-irradiated dorsal skin tissue of mice were prepared by modifying a previously reported method [19]. The tissue sections were stained using three staining reagents, namely H&E (Sigma-Aldrich, St. Louis, MO, USA), Masson’s trichrome (Abcam, Cambridge, UK), and toluidine blue (Sigma-Aldrich) to analyze the microfold number, epidermal thickness, inflammatory and mast cell counts, and collagen fiber-occupied region areas, respectively. Histomorphometry was performed using an Eclipse 80i microscope (Nikon, Tokyo, Japan) equipped with iSolution FL (Ver. 9.1) software (iSolutions Inc., Burnaby, BC, Canada).

### 4.5. Matrix Metalloproteinase (MMP) and HA Determination

A part of the skin biopsy samples was lysed using an EzRIPA lysis kit (Atto, Tokyo, Japan), containing RIPA buffer, protease inhibitor, and phosphatase inhibitor. To determine MMP-2 and MMP-9 levels in the skin tissue extract, gelatin zymography was performed in accordance with the protocol published in a previous report [23]. Briefly, a 30-μg protein sample was mixed with 2% sodium dodecyl sulfate (SDS; Biosesang, Seongnam, Republic of Korea) and resolved on a 10% SDS gel containing 1% gelatin (Koma Biotech, Seoul, Republic of Korea). After washing with 2.5% Triton X-100, the gel was incubated with 50 mM Tris-HCl buffer (pH 7.5), containing 10 mM CaCl_2_ and 0.15 M NaCl, and visualized using Coomassie brilliant blue R-250 (Biosesang) followed by detaining with a mixture of 30% methanol and 10% acetic acid. To determine the level of HA, a 30-μg protein sample was analyzed using a human hyaluronan ELISA kit (R&D Systems, Minneapolis, MN, USA) and the HA concentration was expressed as μg HA/mg protein sample.

### 4.6. Statistical Analysis

Results are expressed as the mean ± standard error (SE), and significant differences were evaluated using a one-way ANOVA. Post-hoc analysis was conducted using the Duncan test, and a p-value of <0.05 was considered significant. Statistical analyses were performed using PASW Statistics 18 (IBM Co., Armonk, NY, USA).

## 5. Conclusions

In the present study, the effect of the oral administration of OBS-E on skin function improvement was investigated in a UVB-irradiated hairless mouse model. Compared with only UVB-irradiated control mice, OBS-E treated mice exhibited decreased wrinkle formation in terms of area, number, length, and depth via OBS-E‒mediated inhibition of MMP-2 and MMP-9 expression, and improved skin hydration and elasticity via OBS-E‒induced increase in elasticity, HA synthesis, and reduction in TEWL. In addition, the oral administration of OBS-E was associated with significant decreases in microfold number, epidermal thickness, and collagen-occupied region area, indicating improvement in skin barrier function, but also in inflammatory cells and mast cells, indicating anti-inflammatory activity. These results demonstrate that OBS-E can effectively enhance skin function in terms of ameliorating wrinkle formation, promoting skin moisturizing, enhancing skin barrier function, and inhibiting inflammatory responses. The obtained results provide a good starting point for the continuation in the process of developing new inner beauty products based on OBS-E.

## Data Availability

No new data were created or analyzed in this study. Data sharing is not applicable to this article.

## Supplementary Materials

The following are available online at https://www.mdpi.com/1424-8247/14/3/222/s1. Figure S1: Body weight changes in the experimental period. G1, non-irradiated control group administered saline; G2, UVB-irradiated control group administered saline; G3 and G4, UVB-irradiated test agent groups administered OBS-E at doses of 50 and 200 mg/kg BW/day, respectively.
