# Supplementary material for: Effect of the Oral Administration of Common Evening Primrose Sprout (Oenothera biennis L.) Extract on Skin Function Improvement in UVB-irradiated Hairless Mice
Source: Pharmaceuticals (Basel). 2021 Mar 6;14(3):222. doi: 10.3390/ph14030222 (PMC8000621; doi:10.3390/ph14030222)
Supplement: Supplementary file 1 [file pharmaceuticals-14-00222-s001.pdf]

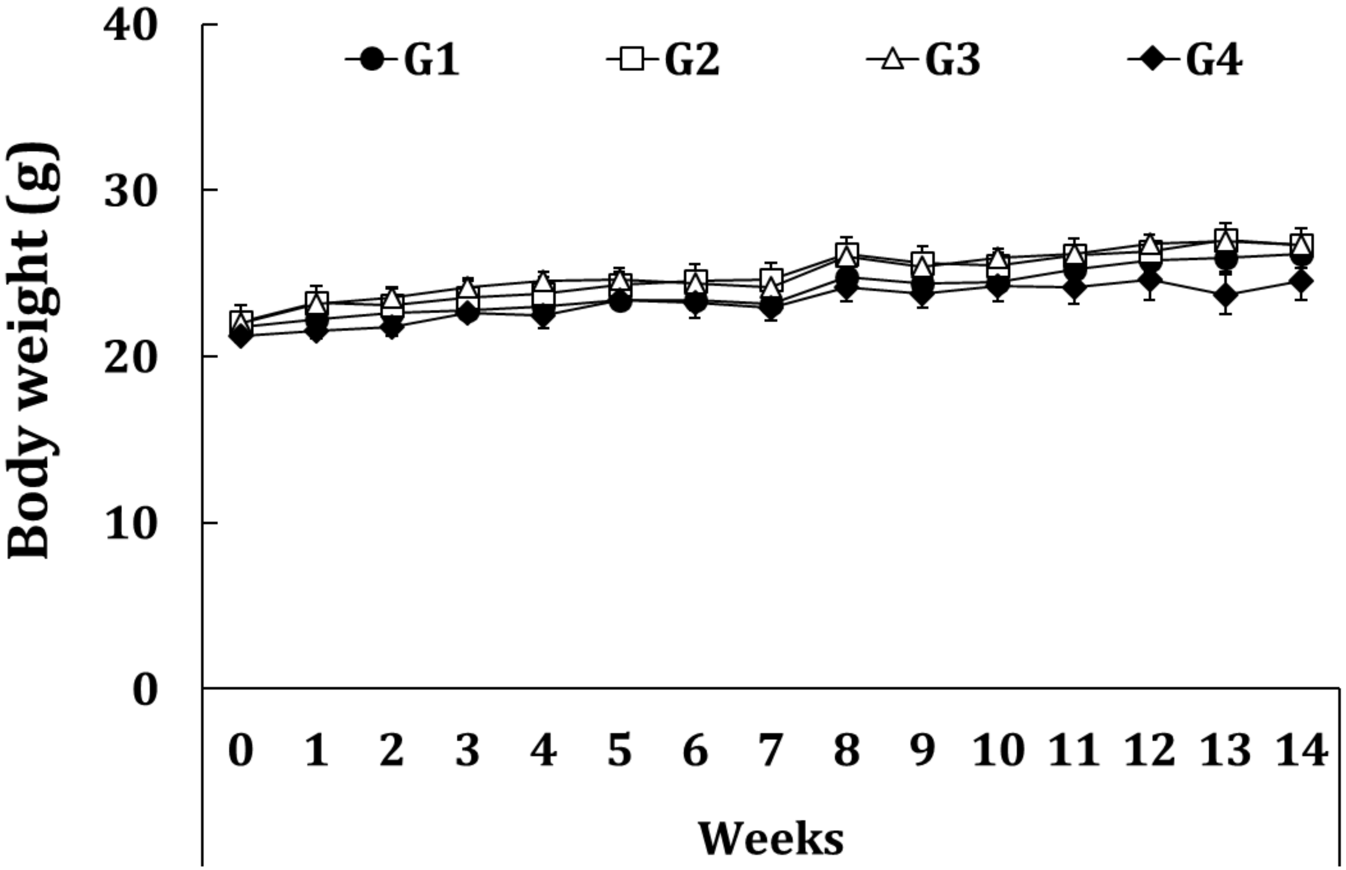

Supplementary Figure 1. Body weight changes in the experimental period. G1, non-irradiated control group administered saline; G2, UVB-irradiated control group administered saline; G3 and G4, UVB-irradiated test agent groups administered OBS-E at doses of 50 and 200 mg/kg BW/day, respectively
